# Evaluation of Equitable Racial and Ethnic Representation Among Departmental Chairs in Academic Medicine, 1980-2019

**DOI:** 10.1001/jamanetworkopen.2021.10726

**Published:** 2021-05-19

**Authors:** Bismarck C. Odei, Reshma Jagsi, Dayssy Alexandra Diaz, Daniel Addison, Andrea Arnett, James B. Odei, Darrion Mitchell

**Affiliations:** 1 Ohio State University James Cancer Center, Columbus; 2 Department of Radiation Oncology, University of Michigan, Ann Arbor; 3 Department of Cardiology, Ohio State University, Columbus; 4 Ohio State University College of Public Health, Columbus

## Abstract

This cross-sectional study evaluates racial and ethnic representation among departmental chairs and faculty in academic medicine in the US from 1980 to 2019.

## Introduction

Diversity in academic leadership is critical in fostering innovative solutions to dynamic challenges in medicine and meeting the needs of an increasingly diverse workforce.^1^ Prior studies^2^ have documented underrepresentation of some racial and ethnic groups in academia and in the departmental chair position; however, it is unknown whether these disparities persist currently and whether they are reflected similarly within individual specialties. Consequently, this study evaluates equitable racial and ethnic diversity among departmental chairs and evaluates the racial/ethnic composition of faculty in academia, which is the pipeline for medical leadership.

## Methods

This study was deemed exempt by the Ohio State University institutional review board, which also granted a waiver of informed consent because the data are publicly available and deidentified, in accordance with 45 CFR §46. This study follows the Strengthening the Reporting of Observational Studies in Epidemiology (STROBE) reporting guideline.

We obtained data from the Association of American Medical Colleges on faculty and departmental chairs for a cross-sectional study. Racial and ethnic designations in these data were self-reported and consistent with the US Census classifications. The racial and ethnic categories examined were Black, Hispanic, Asian (including Pacific Islander and Native Hawaiian), and White. We examined the racial and ethnic composition of medical school faculty, senior faculty (associate and full professors), and chairpersons from 1980 through 2019.

Statistical analysis using SAS statistical software version 9.4 (SAS Institute) assessed whether racial and ethnic representation among faculty was proportional to the racial and ethnic representation among chairs within medical specialties in 2019. The threshold for statistical significance was *P* < .05, using a 2-tailed, 2-sample proportion test. Data analysis was performed from November to December 2020.

## Results

The study examined data representing all faculty from 1980 (cohort size, 53 376 individuals) through 2019 (cohort size, 185 649 individuals), including all departmental chairs (3184 chairs in 1980 and 3311 chairs in 2019), from 122 (in 1980) to 153 (in 2019) MD-granting schools of medicine. Between 1980 and 2019, the proportion of Black, Hispanic, and Asian individuals among all faculty, senior faculty, and departmental chairs remained low compared with White cohorts (Figure).

**Figure.**
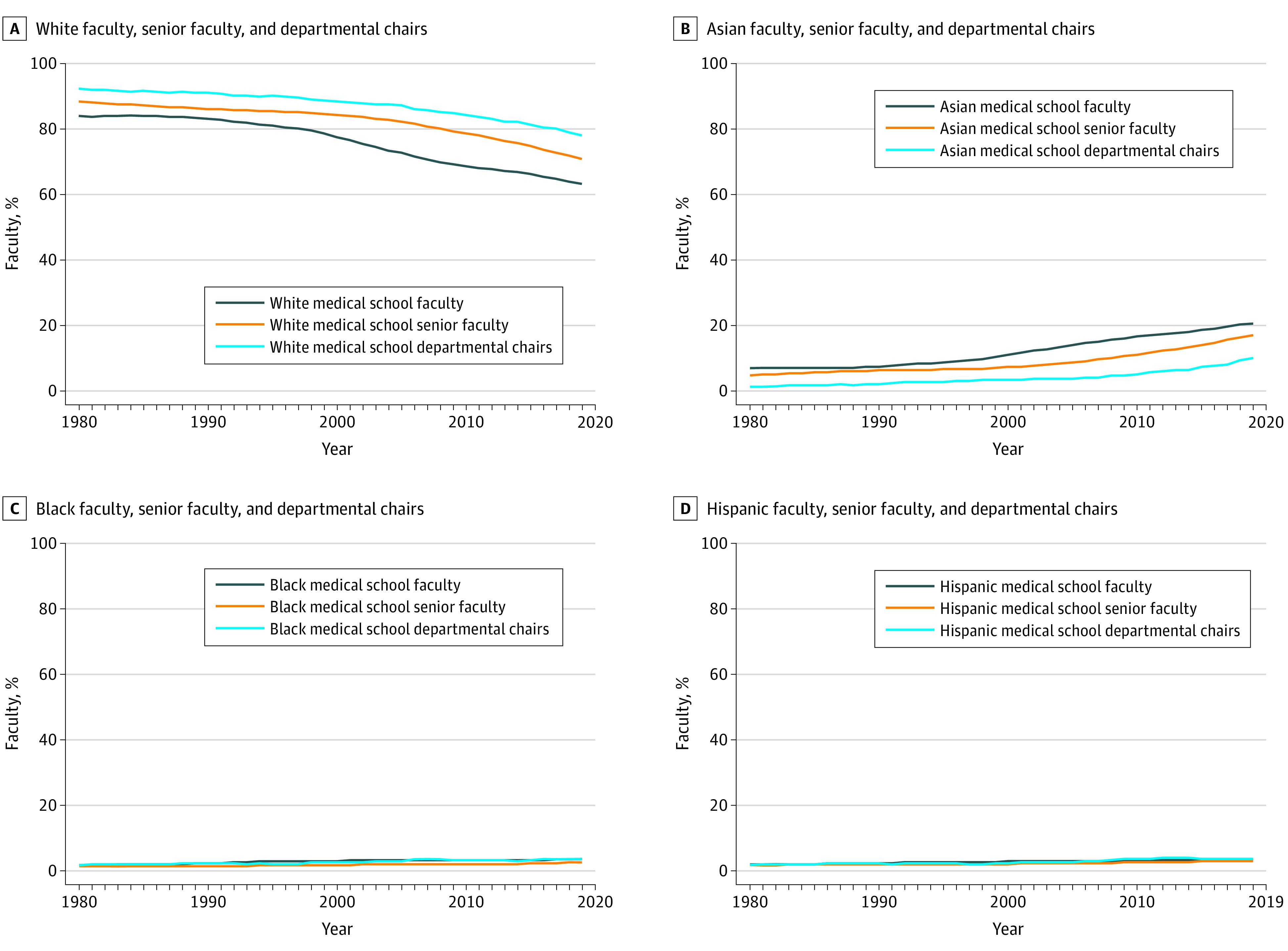
Medical School Faculty, Senior Faculty, and Departmental Chairs by Race/Ethnicity, 1980-2019 In 1980, the total cohort size was 53 376 individuals; in 2019, the total cohort size was 185 649 individuals. A, In 1980, there were 44 881 (84.1%) White faculty, 24 386 (88.5%) White senior faculty (associate and full professors), and 2249 (92.4%) White departmental chairs in MD-granting medical schools. In 2019, there were 117 512 (63.3%) White faculty, 54 622 (70.9%) White senior faculty (associate and full professors), and 2586 (78.1%) White departmental chairs in MD-granting medical schools. B, In 1980, there were 3754 (7.0%) Asian faculty, 1330 (4.8%) Asian senior faculty (associate and full professors), and 33 (1.4%) Asian departmental chairs in MD-granting medical schools. In 2019, there were 38 112 (20.5%) Asian faculty, 13 107 (17.0%) Asian senior faculty (associate and full professors), and 335 (10.1%) Asian departmental chairs in MD-granting medical schools. C, In 1980, there were 993 (1.9%) Black faculty, 411 (1.5%) Black senior faculty (associate and full professors), and 44 (1.8%) Black departmental chairs in MD-granting medical schools. In 2019, there were 6859 (3.7%) Black faculty, 2054 (2.7%) Black senior faculty (associate and full professors), and 124 (3.7%) Black departmental chairs in MD-granting medical schools. D, In 1980, there were 1095 (2.1%) Hispanic faculty, 485 (1.8%) Hispanic senior faculty (associate and full professors), and 45 (1.8%) Hispanic departmental chairs in MD-granting medical schools. In 2019, there were 6275 (3.4%) Hispanic faculty, 2265 (2.9%) Hispanic senior faculty (associate and full professors), and 118 (3.6%) Hispanic departmental chairs in MD-granting medical schools.

Among the represented specialties in 2019 (Table), White individuals were overrepresented in departmental chair positions across all specialties compared with the proportion of White faculty, except in family medicine. Conversely, Asian individuals were underrepresented as departmental chairs across all specialties except in otolaryngology, psychiatry, and dermatology. Among Black and Hispanic individuals, there was proportional representation in departmental chair positions among all specialties, except in family medicine, where there was overrepresentation of Black individuals.

**Table.** Clinical Faculty and Departmental Chairs by Race/Ethnicity and Specialty in 2019

Specialty	Individuals, No. (%) (N = 134 820)^a^											
	White			Asian			Black			Hispanic		
	Faculty	Chairs	*P* value^b^	Faculty	Chairs	*P* value^b^	Faculty	Chairs	*P* value^b^	Faculty	Chairs	*P* value^b^
Anesthesiology	5798 (64.0)	105 (87.5)	<.001	1789 (19.8)	5 (4.2)	<.001	380 (4.2)	2 (1.7)	.25	250 (2.8)	2 (1.7)	.65
Dermatology	995 (65.5)	66 (79.5)	.01	333 (21.9)	10 (12.0)	.05	41 (2.7)	2 (2.4)	>.99	44 (2.9)	4 (4.8)	.50
Emergency medicine	4055 (72.8)	99 (86.8)	.002	687 (12.3)	6 (5.3)	.03	253 (4.5)	4 (3.5)	.77	180 (3.2)	3 (2.6)	.93
Family medicine	3870 (68.3)	105 (76.6)	.05	665 (11.7)	6 (4.4)	.02	329 (5.8)	16 (11.7)	.007	242 (4.3)	6 (4.4)	>.99
Internal medicine	25 543 (59.1)	136 (76.0)	<.001	10 781 (24.9)	20 (11.2)	<.001	1568 (3.6)	9 (5.0)	.42	1481 (3.4)	9 (5.0)	.33
Neurology	3934 (63.5)	101 (78.9)	<.001	1376 (22.2)	13 (10.2)	.002	135 (2.2)	1 (0.8)	.44	169 (2.7)	5 (3.9)	.59
Obstetrics and gynecology	4308 (66.6)	123 (78.8)	.002	834 (12.9)	10 (6.4)	.03	528 (8.2)	10 (6.4)	.52	301 (4.7)	8 (5.1)	.93
Ophthalmology	1844 (60.3)	86 (81.1)	<.001	848 (27.7)	12 (11.3)	<.001	70 (2.3)	4 (3.8)	.50	71 (2.3)	4 (3.8)	.52
Orthopedic surgery	3107 (75.3)	107 (89.2)	<.001	551 (13.4)	5 (4.2)	.005	103 (2.5)	4 (3.3)	.78	85 (2.1)	2 (1.7)	>.99
Otolaryngology	1538 (68.8)	70 (80.5)	.03	409 (18.3)	12 (13.8)	.35	42 (1.9)	1 (1.1)	.93	66 (3.0)	3 (3.4)	>.99
Pediatrics	15 371 (65.2)	119 (77.3)	.002	4166 (17.7)	11 (7.1)	<.001	984 (4.2)	7 (4.5)	.98	912 (3.9)	8 (5.2)	.52
Psychiatry	7675 (68.6)	121 (78.1)	.02	1357 (12.1)	12 (7.7)	.12	429 (3.8)	4 (2.6)	.55	421 (3.8)	9 (5.8)	.20
Radiology	5946 (60.1)	160 (74.8)	<.001	2671 (27.0)	34 (15.9)	<.001	229 (2.3)	9 (4.2)	.11	237 (2.4)	5 (2.3)	>.99
Surgery (general)	10 821 (65.7)	293 (77.2)	<.001	2889 (17.8)	48 (11.5)	.01	575 (3.5)	14 (3.7)	>.99	534 (3.3)	18 (4.7)	.16

^a^ Data are shown for a subset of 14 specialties, not the entire cohort.

^b^ *P* values that are significant denote overrepresentation or underrepresentation of departmental chairs of a specific race/ethnicity within a given specialty. *P* values that are not statistically significant denote proportional representation of departmental chairs relative to faculty of a specific race/ethnicity within a given specialty.

## Discussion

Our study revealed some gains in the racial and ethnic composition of chairs over time, although White individuals remained the predominant group. Particularly striking was the significant underrepresentation of Asian individuals among departmental chairs compared with their representation among faculty more generally. Asian individuals appear to have joined medical faculty at a level that exceeds their representation in the general population. Thus, their designation as a non-underrepresented group has likely contributed to their deprioritization in initiatives to enhance diversity in the upper echelons of medicine.^3^ This trend of high representation of Asian individuals in professional settings with subsequent limitations in upward mobility toward leadership roles appears to be cross-disciplinary and has also been documented in business and law institutions,^3^ suggesting possible pervasive implicit bias. Efforts to reverse these trends require diversity initiatives that acknowledge these realities and prioritize the elimination of barriers to medical leadership among Asian faculty.

In contrast to Asian individuals, Black and Hispanic individuals continue to be persistently underrepresented in medicine and in faculty positions, suggesting a need for bolder and more transformational pipeline interventions.^4,5^ Although our study appears to show no further obvious decrement in Black and Hispanic individuals reaching departmental chair positions once on faculty, the low proportion of underrepresented racial/ethnic groups among faculty and chairs may mask further evidence of underrepresentation of chairs from these racial/ethnic groups due to the floor effect. Given the observations in the analysis of Asian individuals, ongoing vigilance is needed to ensure that increased faculty representation translates into increased representation among leaders.

A limitation of this study is that the Association of American Medical Colleges data has minor inaccuracies in the number of reported faculty; however, the proportion of faculty across key variables remains generally accurate.^6^ In addition, the degree of heterogeneity among certain racial/ethnic groups may be underappreciated in our data set.

Chair search committees may benefit from increased group diversity and more formal orientation about the merits of diverse leadership in academic medicine. In addition, early and strategically paired mentorship of underrepresented racial/ethnic faculty and Asian faculty with seasoned academic leaders appears to be increasingly crucial and may provide the underpinning for career development and eventual acceleration of racial and ethnic diversity in academic leadership.
